# 2-Amino-4-(2-chloro­phen­yl)-7,7-di­methyl-5-oxo-5,6,7,8-tetra­hydro-4*H*-chromene-3-carbonitrile hemihydrate

**DOI:** 10.1107/S1600536812003807

**Published:** 2012-02-24

**Authors:** Xiao-Lei Hu, Zhong-Xia Wang, Fang-Ming Wang, Guang-Fan Han

**Affiliations:** a School of Biology and Chemical Engineering, Jiangsu University of Science and Technology, Zhenjiang 212003, People’s Republic of China

## Abstract

The asymmetric unit of the title compound, C_18_H_17_ClN_2_O_2_·0.5H_2_O, contains two organic mol­ecules and one solvent water mol­ecule. In each organic mol­ecule, the cyclo­hexene ring adopts an envelope conformation with the C atom connecting the two methyl groups on the flap; the 4*H*-pyran ring is nearly planar [maximum deviation = 0.113 (3) Å in one mol­ecule and 0.089 (3) Å in the other mol­ecule] and is approximately perpendicular to the chloro­phenyl ring [dihedral angle = 86.43 (15)° in one mol­ecule and 89.73 (15)° in the other mol­ecule]. Inter­molecular N—H⋯N, N—H⋯O, O—H⋯O and O—H⋯Cl hydrogen bonding is present in the crystal.

## Related literature

For background to 2-amino-4*H*-benzopyran-3-carbonitriles, see: Gao *et al.* (2001 ▶); Xu *et al.* (2011 ▶); Luan *et al.* (2011 ▶). For the synthesis of 2-amino-4*H*-benzopyran-3-carbonitriles, see: Shi *et al.* (2003 ▶); Bao *et al.* (2007 ▶).
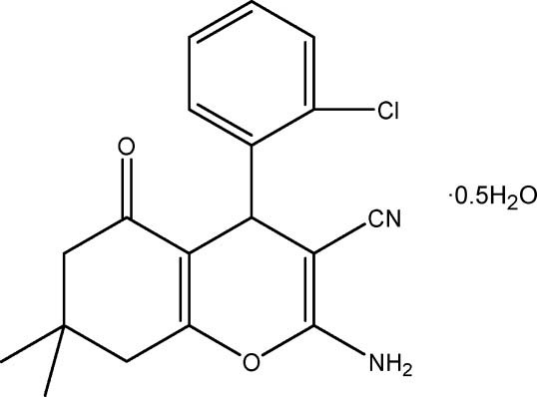


## Experimental

### 

#### Crystal data

C_18_H_17_ClN_2_O_2_·0.5H_2_O
*M* *_r_* = 337.80
Monoclinic, 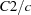

*a* = 31.431 (6) Å
*b* = 9.3230 (19) Å
*c* = 25.079 (5) Å
β = 111.24 (3)°
*V* = 6850 (2) Å^3^
*Z* = 16
Mo *K*α radiation
μ = 0.24 mm^−1^
*T* = 291 K
0.20 × 0.20 × 0.20 mm

#### Data collection

Bruker SMART APEX CCD diffractometer
Absorption correction: multi-scan (*SADABS*; Bruker, 2001 ▶) *T*_min_ = 0.945, *T*_max_ = 0.959
15314 measured reflections
6243 independent reflections
3806 reflections with *I* > 2σ(*I*)
*R*_int_ = 0.054

#### Refinement

*R*[*F* ^2^ > 2σ(*F* ^2^)] = 0.057
*wR*(*F* ^2^) = 0.168
*S* = 1.00
6243 reflections
428 parameters
H-atom parameters constrained
Δρ_max_ = 0.36 e Å^−3^
Δρ_min_ = −0.50 e Å^−3^

### 

Data collection: *SMART* (Bruker, 2007 ▶); cell refinement: *SAINT* (Bruker, 2007 ▶); data reduction: *SAINT*; program(s) used to solve structure: *SHELXTL* (Sheldrick, 2008 ▶); program(s) used to refine structure: *SHELXTL*; molecular graphics: *SHELXTL*; software used to prepare material for publication: *SHELXTL*.

## Supplementary Material

Crystal structure: contains datablock(s) I, global. DOI: 10.1107/S1600536812003807/xu5439sup1.cif

Structure factors: contains datablock(s) I. DOI: 10.1107/S1600536812003807/xu5439Isup2.hkl

Supplementary material file. DOI: 10.1107/S1600536812003807/xu5439Isup3.cml

Additional supplementary materials:  crystallographic information; 3D view; checkCIF report

## Figures and Tables

**Table 1:** Hydrogen-bond geometry (Å, °)

*D*—H⋯*A*	*D*—H	H⋯*A*	*D*⋯*A*	*D*—H⋯*A*
N2—H2*A*⋯N3^i^	0.86	2.11	2.957 (4)	167
N2—H2*B*⋯O1*W*^ii^	0.86	2.01	2.793 (4)	152
N4—H4*A*⋯N1^iii^	0.86	2.20	3.048 (4)	169
N4—H4*B*⋯O2^iv^	0.86	2.13	2.957 (3)	161
O1*W*—H1*X*⋯O4	0.96	1.95	2.754 (4)	139
O1*W*—H1*Y*⋯Cl2	0.96	2.29	3.168 (4)	151

Symmetry codes: (i) 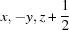; (ii) 
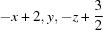
; (iii) 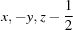; (iv) 
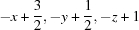
.

**Fig. 1.**
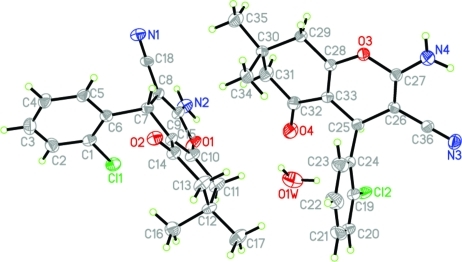
The molecular structure of the title compound, showing 30% probability displacement and the atom numbering scheme.

**Fig. 2.**
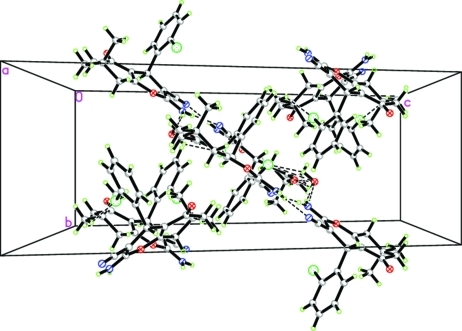
Packing diagram of the title compound viewed along the a axis. H-bonding interactions are shown as dashed lines.

## supplementary crystallographic information

### Comment

2-Amino-4*H*-benzopyran-3-carbonitriles have attracted strong interest because of their wide biological activities, such as anti-anaphylaxis, anti-achondroplasty and anti-cancer activity (Gao *et al.*, 2001). They also have potential application in the treatment of psoriatic arthritis and rheumatoid (Xu *et al.*, 2011). Furthermore, existence of amino group and cyano group make it a useful building block for organic transformations (Luan *et al.*, 2011). Many methods have been reported for synthesis of 2-amino-4*H*-benzopyran-3-carbonitriles (Shi *et al.*, 2003; Bao *et al.*, 2007). Herein we report the synthesis and crystal structure of 2-amino-5,6,7,8-tetrahydro-5-oxo-4-(2-chloro-phenyl)-7,7-dimethyl-4*H*-benzo[*b*]pyran-3-carbonitrile.

The molecular structure of the title compound is shown in Fig. 1. A nd it packing diagram is shown in Fig. 2. As shown in Fig. 1,there include two isomer molecules and one dissociated water molecule in an asymmetric unit of the title compound. And all their cyclohexyl rings show in half-boat conformations. The dihedral angles between the benzene plane and the cyclohexanone plane in each molecular structures are 75.07 (12)° and 78.25 (11)°. The four kinds of intermolecular hydrogen bonds in the crystal, such as N—H···N, N—H···O, O—H···O and O—H···Cl, link all molecules into a three-dimensional supramolecular structure.

### Experimental

A mixture of 2-chlorobenzaldehyde (5 mmol) and malononitrile (5 mmol) in ethanol (10 ml) were stirred at 353 K for 2 h, using K_2_CO_3_ as catalyst. Then the reaction crude were cooled to room temperature and slowly added cold water (30 ml) with continuously stirring. The solid was filtrated, and recrystallized from ethanol. Then it was reacted with 5,5-dimethyl-1,3-cyclohexanedione (5 mmol) in glycol (10 ml) at 353 K for 4 h. The mixture was cooled and added cold water again. The solid was filtrated, and recrystallized from ethanol, yield 78%. Single crystal of the title compound suitable for X-ray analysis were obtained by evaporating from methanol/water solution at room temperature for 3 weeks.

### Refinement

All H atoms were fixed geometrically and treated as riding with C—H = 0.93–0.98 Å, N–H = 0.86 and O–H = 0.96 Å, and refined in riding mode with *U*_iso_(H) = 1.5*U*_eq_(C) for methyl H atoms and 1.2*U*_eq_(C,*N*,*O*) for the others.

### Crystal data

**Table:**

C_18_H_17_ClN_2_O_2_·0.5H_2_O	*F*(000) = 2832
*M**_r_* = 337.80	*D*_x_ = 1.310 Mg m^−^^3^
Monoclinic, *C*2/*c*	Mo *K*α radiation, λ = 0.71073 Å
Hall symbol: -C 2yc	Cell parameters from 3715 reflections
*a* = 31.431 (6) Å	θ = 2.1–23.9°
*b* = 9.3230 (19) Å	µ = 0.24 mm^−^^1^
*c* = 25.079 (5) Å	*T* = 291 K
β = 111.24 (3)°	Prism, colorless
*V* = 6850 (2) Å^3^	0.20 × 0.20 × 0.20 mm
*Z* = 16	

### Data collection

**Table:**

Bruker SMART APEX CCD diffractometer	6243 independent reflections
Radiation source: sealed tube	3806 reflections with *I* > 2σ(*I*)
Graphite monochromator	*R*_int_ = 0.054
φ and ω scans	θ_max_ = 25.4°, θ_min_ = 3.1°
Absorption correction: multi-scan (*SADABS*; Bruker, 2001)	*h* = −37→31
*T*_min_ = 0.945, *T*_max_ = 0.959	*k* = −9→11
15314 measured reflections	*l* = −25→30

### Refinement

**Table:**

Refinement on *F*^2^	Primary atom site location: structure-invariant direct methods
Least-squares matrix: full	Secondary atom site location: difference Fourier map
*R*[*F*^2^ > 2σ(*F*^2^)] = 0.057	Hydrogen site location: inferred from neighbouring sites
*wR*(*F*^2^) = 0.168	H-atom parameters constrained
*S* = 1.00	*w* = 1/[σ^2^(*F*_o_^2^) + (0.0954*P*)^2^] where *P* = (*F*_o_^2^ + 2*F*_c_^2^)/3
6243 reflections	(Δ/σ)_max_ < 0.001
428 parameters	Δρ_max_ = 0.36 e Å^−^^3^
0 restraints	Δρ_min_ = −0.50 e Å^−^^3^

### Special details

**Table:**

Experimental. Least-squares planes (*x*,*y*,*z* in crystal coordinates) and deviations from them (* indicates atom used to define plane)- 12.7873 (0.0387) *x* - 4.5876 (0.0107) *y* + 21.6869 (0.0196) *z* = 2.4952 (0.0356)* -0.0054 (0.0022) C1 * 0.0083 (0.0024) C2 * -0.0071 (0.0025) C3 * 0.0030 (0.0023) C4 * -0.0001 (0.0020) C5 * 0.0014 (0.0020) C6Rms deviation of fitted atoms = 0.005211.1322 (0.0507) *x* + 7.9044 (0.0087) *y* + 6.0068 (0.0449) *z* = 18.4619 (0.0327)Angle to previous plane (with approximate e.s.d.) = 75.07 (12)* -0.0232 (0.0025) C10 * 0.0250 (0.0019) C11 * -0.0262 (0.0019) C13 * 0.0290 (0.0024) C14 * -0.0045 (0.0022) C15Rms deviation of fitted atoms = 0.0233- 6.9377 (0.0455) *x* + 5.2108 (0.0111) *y* + 20.6895 (0.0225) *z* = 5.0012 (0.0397)Angle to previous plane (with approximate e.s.d.) = 44.67 (16)* -0.0049 (0.0022) C19 * 0.0034 (0.0025) C20 * 0.0014 (0.0027) C21 * -0.0045 (0.0027) C22 * 0.0029 (0.0025) C23 * 0.0018 (0.0022) C24Rms deviation of fitted atoms = 0.00341.7021 (0.0460) *x* - 8.5297 (0.0071) *y* + 8.8582 (0.0381) *z* = 6.0421 (0.0321)Angle to previous plane (with approximate e.s.d.) = 78.25 (11)* 0.0432 (0.0021) C28 * -0.0437 (0.0016) C29 * 0.0442 (0.0017) C31 * -0.0470 (0.0023) C32 * 0.0032 (0.0020) C33Rms deviation of fitted atoms = 0.0399
Geometry. All e.s.d.'s (except the e.s.d. in the dihedral angle between two l.s. planes) are estimated using the full covariance matrix. The cell e.s.d.'s are taken into account individually in the estimation of e.s.d.'s in distances, angles and torsion angles; correlations between e.s.d.'s in cell parameters are only used when they are defined by crystal symmetry. An approximate (isotropic) treatment of cell e.s.d.'s is used for estimating e.s.d.'s involving l.s. planes.
Refinement. Refinement of *F*^2^ against ALL reflections. The weighted *R*-factor *wR* and goodness of fit *S* are based on *F*^2^, conventional *R*-factors *R* are based on *F*, with *F* set to zero for negative *F*^2^. The threshold expression of *F*^2^ > σ(*F*^2^) is used only for calculating *R*-factors(gt) *etc*. and is not relevant to the choice of reflections for refinement. *R*-factors based on *F*^2^ are statistically about twice as large as those based on *F*, and *R*- factors based on ALL data will be even larger.

### Fractional atomic coordinates and isotropic or equivalent isotropic displacement parameters (Å^2^)

**Table:**

	*x*	*y*	*z*	*U*_iso_*/*U*_eq_	
C1	0.87769 (10)	0.7977 (4)	0.80107 (13)	0.0404 (7)	
C2	0.87215 (12)	0.9275 (4)	0.82590 (15)	0.0495 (8)	
H2	0.8973	0.9748	0.8516	0.059*	
C3	0.82914 (13)	0.9840 (4)	0.81178 (15)	0.0531 (9)	
H3	0.8251	1.0715	0.8271	0.064*	
C4	0.79199 (11)	0.9116 (3)	0.77502 (14)	0.0453 (8)	
H4	0.7629	0.9499	0.7664	0.054*	
C5	0.79701 (11)	0.7834 (3)	0.75071 (13)	0.0395 (7)	
H5	0.7714	0.7367	0.7256	0.047*	
C6	0.84100 (10)	0.7219 (3)	0.76370 (12)	0.0335 (6)	
C7	0.84471 (9)	0.5760 (3)	0.73737 (12)	0.0319 (6)	
H7	0.8135	0.5475	0.7140	0.038*	
C8	0.86352 (9)	0.4584 (3)	0.78183 (12)	0.0322 (6)	
C9	0.90599 (10)	0.3998 (3)	0.79355 (12)	0.0379 (7)	
C10	0.91149 (10)	0.5090 (4)	0.71063 (12)	0.0405 (7)	
C11	0.93956 (11)	0.5026 (4)	0.67499 (15)	0.0499 (9)	
H11A	0.9348	0.4110	0.6554	0.060*	
H11B	0.9715	0.5087	0.6994	0.060*	
C12	0.92855 (10)	0.6222 (4)	0.63111 (13)	0.0395 (7)	
C13	0.87805 (11)	0.6376 (5)	0.60281 (15)	0.0527 (9)	
H13A	0.8664	0.5554	0.5782	0.063*	
H13B	0.8717	0.7223	0.5787	0.063*	
C14	0.85235 (10)	0.6499 (3)	0.64347 (13)	0.0381 (7)	
C15	0.87098 (9)	0.5780 (3)	0.69802 (12)	0.0335 (6)	
C16	0.94814 (13)	0.7637 (4)	0.66638 (15)	0.0546 (9)	
H16A	0.9448	0.8423	0.6405	0.082*	
H16B	0.9799	0.7504	0.6890	0.082*	
H16C	0.9316	0.7840	0.6910	0.082*	
C17	0.95296 (13)	0.6054 (5)	0.59000 (14)	0.0586 (10)	
H17A	0.9409	0.5238	0.5659	0.088*	
H17B	0.9850	0.5916	0.6109	0.088*	
H17C	0.9486	0.6900	0.5668	0.088*	
C18	0.83765 (10)	0.4130 (3)	0.81353 (13)	0.0365 (7)	
C19	0.94532 (11)	0.1569 (4)	0.51895 (14)	0.0454 (8)	
C20	0.96005 (16)	0.2732 (4)	0.49501 (17)	0.0634 (11)	
H20	0.9910	0.2864	0.5024	0.076*	
C21	0.92824 (18)	0.3689 (4)	0.46015 (17)	0.0686 (12)	
H21	0.9379	0.4462	0.4440	0.082*	
C22	0.88321 (17)	0.3500 (5)	0.44952 (17)	0.0701 (12)	
H22	0.8619	0.4138	0.4259	0.084*	
C23	0.86889 (13)	0.2357 (4)	0.47387 (15)	0.0539 (9)	
H23	0.8379	0.2247	0.4665	0.065*	
C24	0.89882 (10)	0.1381 (3)	0.50843 (12)	0.0349 (6)	
C25	0.88120 (9)	0.0083 (3)	0.53129 (11)	0.0324 (6)	
H25	0.9078	−0.0417	0.5581	0.039*	
C26	0.85659 (9)	−0.0962 (3)	0.48349 (11)	0.0319 (6)	
C27	0.81138 (9)	−0.1227 (3)	0.46640 (12)	0.0349 (6)	
C28	0.80578 (9)	0.0119 (3)	0.54358 (12)	0.0333 (6)	
C29	0.77329 (10)	0.0455 (3)	0.57239 (14)	0.0376 (7)	
H29A	0.7681	−0.0404	0.5910	0.045*	
H29B	0.7443	0.0739	0.5438	0.045*	
C30	0.79024 (10)	0.1653 (3)	0.61691 (12)	0.0359 (7)	
C31	0.83979 (12)	0.1330 (4)	0.65379 (13)	0.0514 (9)	
H31A	0.8517	0.2110	0.6807	0.062*	
H31B	0.8409	0.0464	0.6757	0.062*	
C32	0.86993 (11)	0.1136 (4)	0.61901 (13)	0.0422 (7)	
C33	0.85047 (10)	0.0454 (3)	0.56274 (12)	0.0347 (6)	
C34	0.78655 (12)	0.3096 (3)	0.58672 (15)	0.0493 (8)	
H34A	0.8034	0.3059	0.5616	0.074*	
H34B	0.7989	0.3837	0.6147	0.074*	
H34C	0.7551	0.3299	0.5649	0.074*	
C35	0.76102 (14)	0.1704 (4)	0.65389 (17)	0.0600 (10)	
H35A	0.7717	0.2460	0.6816	0.090*	
H35B	0.7632	0.0804	0.6733	0.090*	
H35C	0.7298	0.1881	0.6301	0.090*	
C36	0.88196 (9)	−0.1639 (3)	0.45490 (12)	0.0341 (6)	
Cl1	0.93301 (3)	0.73422 (10)	0.82028 (4)	0.0497 (2)	
Cl2	0.98615 (3)	0.04182 (10)	0.56223 (4)	0.0535 (3)	
N1	0.81581 (8)	0.3730 (3)	0.83876 (11)	0.0437 (7)	
N2	0.92876 (9)	0.3135 (3)	0.83506 (11)	0.0517 (7)	
H2A	0.9171	0.2864	0.8596	0.062*	
H2B	0.9553	0.2836	0.8378	0.062*	
N3	0.90360 (9)	−0.2218 (3)	0.43262 (11)	0.0510 (7)	
N4	0.78563 (9)	−0.2024 (3)	0.42255 (12)	0.0490 (7)	
H4A	0.7979	−0.2469	0.4018	0.059*	
H4B	0.7569	−0.2109	0.4154	0.059*	
O1	0.93091 (7)	0.4299 (3)	0.75980 (9)	0.0469 (6)	
O2	0.81516 (6)	0.7103 (2)	0.62866 (8)	0.0404 (5)	
O3	0.78475 (6)	−0.0620 (2)	0.49361 (9)	0.0394 (5)	
O4	0.91002 (8)	0.1490 (3)	0.63733 (10)	0.0601 (7)	
O1W	1.00378 (11)	0.1570 (4)	0.68730 (14)	0.0858 (9)	
H1X	0.9740	0.1980	0.6788	0.103*	
H1Y	1.0031	0.0925	0.6572	0.103*	

### Atomic displacement parameters (Å^2^)

**Table:**

	*U*^11^	*U*^22^	*U*^33^	*U*^12^	*U*^13^	*U*^23^
C1	0.0300 (14)	0.0491 (19)	0.0441 (16)	0.0001 (14)	0.0158 (13)	0.0035 (15)
C2	0.058 (2)	0.0449 (18)	0.0470 (19)	−0.0052 (16)	0.0202 (16)	−0.0037 (15)
C3	0.065 (2)	0.0428 (18)	0.056 (2)	0.0084 (17)	0.0263 (18)	−0.0032 (16)
C4	0.0401 (17)	0.0422 (18)	0.057 (2)	0.0121 (14)	0.0216 (16)	0.0047 (16)
C5	0.0461 (17)	0.0383 (16)	0.0427 (16)	0.0056 (14)	0.0264 (14)	0.0090 (14)
C6	0.0379 (15)	0.0331 (15)	0.0311 (14)	0.0011 (13)	0.0144 (12)	0.0079 (12)
C7	0.0257 (13)	0.0305 (14)	0.0398 (16)	0.0047 (11)	0.0122 (12)	0.0083 (12)
C8	0.0358 (15)	0.0309 (15)	0.0332 (15)	0.0051 (12)	0.0165 (12)	0.0032 (12)
C9	0.0427 (16)	0.0400 (17)	0.0365 (16)	0.0123 (13)	0.0212 (13)	0.0135 (13)
C10	0.0361 (15)	0.0556 (19)	0.0319 (15)	0.0101 (14)	0.0147 (12)	0.0134 (14)
C11	0.0471 (18)	0.062 (2)	0.0499 (19)	0.0162 (16)	0.0293 (16)	0.0167 (17)
C12	0.0362 (15)	0.0515 (19)	0.0375 (16)	−0.0008 (14)	0.0213 (13)	0.0072 (14)
C13	0.0431 (18)	0.077 (3)	0.0424 (18)	0.0100 (17)	0.0210 (15)	0.0091 (18)
C14	0.0357 (16)	0.0418 (17)	0.0389 (16)	0.0047 (13)	0.0160 (13)	0.0054 (14)
C15	0.0321 (14)	0.0389 (15)	0.0325 (15)	0.0048 (12)	0.0155 (12)	0.0022 (12)
C16	0.056 (2)	0.058 (2)	0.050 (2)	−0.0085 (17)	0.0193 (17)	−0.0055 (17)
C17	0.058 (2)	0.092 (3)	0.0316 (17)	0.007 (2)	0.0230 (16)	0.0071 (18)
C18	0.0351 (15)	0.0315 (15)	0.0446 (17)	−0.0005 (12)	0.0166 (13)	0.0109 (13)
C19	0.0461 (18)	0.0511 (19)	0.0476 (18)	−0.0146 (15)	0.0274 (15)	−0.0103 (15)
C20	0.089 (3)	0.060 (2)	0.067 (2)	−0.030 (2)	0.059 (2)	−0.014 (2)
C21	0.114 (4)	0.050 (2)	0.058 (2)	−0.032 (2)	0.051 (2)	−0.0052 (19)
C22	0.101 (3)	0.063 (3)	0.048 (2)	−0.023 (2)	0.028 (2)	−0.0009 (19)
C23	0.059 (2)	0.055 (2)	0.0469 (19)	−0.0096 (17)	0.0180 (17)	0.0055 (17)
C24	0.0441 (16)	0.0340 (15)	0.0318 (15)	−0.0084 (13)	0.0199 (13)	−0.0054 (13)
C25	0.0294 (14)	0.0376 (15)	0.0284 (14)	−0.0032 (12)	0.0084 (11)	−0.0006 (12)
C26	0.0340 (14)	0.0311 (15)	0.0313 (14)	−0.0050 (12)	0.0126 (12)	−0.0013 (12)
C27	0.0330 (15)	0.0351 (15)	0.0376 (16)	0.0028 (12)	0.0138 (13)	−0.0013 (13)
C28	0.0307 (14)	0.0344 (15)	0.0374 (15)	−0.0007 (12)	0.0153 (12)	−0.0027 (13)
C29	0.0359 (15)	0.0325 (16)	0.0514 (18)	−0.0008 (12)	0.0242 (14)	−0.0031 (13)
C30	0.0470 (17)	0.0296 (15)	0.0389 (16)	−0.0066 (13)	0.0252 (14)	0.0006 (12)
C31	0.059 (2)	0.064 (2)	0.0319 (17)	0.0008 (17)	0.0175 (16)	−0.0021 (15)
C32	0.0450 (18)	0.0496 (19)	0.0319 (15)	−0.0044 (15)	0.0138 (14)	−0.0021 (14)
C33	0.0375 (15)	0.0338 (15)	0.0373 (15)	−0.0058 (12)	0.0191 (13)	0.0002 (12)
C34	0.065 (2)	0.0283 (16)	0.064 (2)	−0.0015 (14)	0.0340 (18)	−0.0024 (15)
C35	0.071 (2)	0.059 (2)	0.066 (2)	−0.0012 (19)	0.045 (2)	−0.0007 (19)
C36	0.0286 (14)	0.0431 (17)	0.0284 (14)	−0.0043 (12)	0.0076 (12)	−0.0027 (12)
Cl1	0.0312 (4)	0.0594 (5)	0.0549 (5)	0.0005 (3)	0.0112 (3)	0.0006 (4)
Cl2	0.0403 (4)	0.0652 (6)	0.0657 (6)	−0.0023 (4)	0.0321 (4)	0.0019 (4)
N1	0.0330 (13)	0.0491 (16)	0.0484 (16)	0.0086 (12)	0.0141 (12)	0.0181 (13)
N2	0.0412 (14)	0.068 (2)	0.0434 (15)	0.0124 (14)	0.0122 (12)	0.0155 (14)
N3	0.0418 (15)	0.070 (2)	0.0426 (15)	−0.0052 (14)	0.0167 (13)	−0.0163 (14)
N4	0.0450 (15)	0.0532 (17)	0.0496 (16)	−0.0059 (13)	0.0181 (13)	−0.0095 (14)
O1	0.0421 (12)	0.0658 (15)	0.0399 (12)	0.0231 (11)	0.0234 (10)	0.0228 (11)
O2	0.0289 (10)	0.0487 (12)	0.0405 (11)	0.0057 (9)	0.0086 (9)	0.0075 (10)
O3	0.0291 (10)	0.0435 (12)	0.0474 (12)	−0.0045 (9)	0.0159 (9)	−0.0140 (10)
O4	0.0471 (14)	0.0865 (19)	0.0428 (13)	−0.0122 (13)	0.0117 (11)	−0.0204 (13)
O1W	0.0741 (19)	0.089 (2)	0.085 (2)	−0.0176 (17)	0.0176 (17)	0.0101 (18)

### Geometric parameters (Å, º)

**Table:**

C1—C6	1.387 (4)	C20—C21	1.388 (6)
C1—C2	1.401 (5)	C20—H20	0.9300
C1—Cl1	1.732 (3)	C21—C22	1.354 (6)
C2—C3	1.372 (5)	C21—H21	0.9300
C2—H2	0.9300	C22—C23	1.382 (5)
C3—C4	1.375 (5)	C22—H22	0.9300
C3—H3	0.9300	C23—C24	1.369 (5)
C4—C5	1.377 (4)	C23—H23	0.9300
C4—H4	0.9300	C24—C25	1.526 (4)
C5—C6	1.421 (4)	C25—C33	1.492 (4)
C5—H5	0.9300	C25—C26	1.521 (4)
C6—C7	1.535 (4)	C25—H25	0.9800
C7—C15	1.499 (4)	C26—C27	1.350 (4)
C7—C8	1.523 (4)	C26—C36	1.401 (4)
C7—H7	0.9800	C27—N4	1.331 (4)
C8—C9	1.372 (4)	C27—O3	1.379 (3)
C8—C18	1.393 (4)	C28—C33	1.346 (4)
C9—N2	1.305 (4)	C28—O3	1.372 (3)
C9—O1	1.374 (3)	C28—C29	1.482 (4)
C10—C15	1.358 (4)	C29—C30	1.532 (4)
C10—O1	1.376 (3)	C29—H29A	0.9700
C10—C11	1.467 (4)	C29—H29B	0.9700
C11—C12	1.516 (4)	C30—C35	1.524 (4)
C11—H11A	0.9700	C30—C31	1.526 (5)
C11—H11B	0.9700	C30—C34	1.527 (4)
C12—C13	1.493 (4)	C31—C32	1.513 (4)
C12—C17	1.500 (4)	C31—H31A	0.9700
C12—C16	1.583 (5)	C31—H31B	0.9700
C13—C14	1.517 (4)	C32—O4	1.220 (4)
C13—H13A	0.9700	C32—C33	1.464 (4)
C13—H13B	0.9700	C34—H34A	0.9600
C14—O2	1.227 (3)	C34—H34B	0.9600
C14—C15	1.443 (4)	C34—H34C	0.9600
C16—H16A	0.9600	C35—H35A	0.9600
C16—H16B	0.9600	C35—H35B	0.9600
C16—H16C	0.9600	C35—H35C	0.9600
C17—H17A	0.9600	C36—N3	1.158 (4)
C17—H17B	0.9600	N2—H2A	0.8599
C17—H17C	0.9600	N2—H2B	0.8600
C18—N1	1.151 (4)	N4—H4A	0.8600
C19—C20	1.397 (5)	N4—H4B	0.8599
C19—C24	1.399 (4)	O1W—H1X	0.9600
C19—Cl2	1.723 (4)	O1W—H1Y	0.9599
			
C6—C1—C2	122.3 (3)	C19—C20—H20	120.2
C6—C1—Cl1	121.3 (2)	C22—C21—C20	120.3 (4)
C2—C1—Cl1	116.5 (2)	C22—C21—H21	119.9
C3—C2—C1	119.1 (3)	C20—C21—H21	119.9
C3—C2—H2	120.4	C21—C22—C23	119.9 (4)
C1—C2—H2	120.4	C21—C22—H22	120.1
C2—C3—C4	120.2 (3)	C23—C22—H22	120.1
C2—C3—H3	119.9	C24—C23—C22	122.2 (4)
C4—C3—H3	119.9	C24—C23—H23	118.9
C3—C4—C5	121.2 (3)	C22—C23—H23	118.9
C3—C4—H4	119.4	C23—C24—C19	117.9 (3)
C5—C4—H4	119.4	C23—C24—C25	120.3 (3)
C4—C5—C6	120.5 (3)	C19—C24—C25	121.7 (3)
C4—C5—H5	119.8	C33—C25—C26	108.9 (2)
C6—C5—H5	119.8	C33—C25—C24	114.0 (2)
C1—C6—C5	116.8 (3)	C26—C25—C24	111.2 (2)
C1—C6—C7	124.6 (3)	C33—C25—H25	107.5
C5—C6—C7	118.6 (3)	C26—C25—H25	107.5
C15—C7—C8	109.3 (2)	C24—C25—H25	107.5
C15—C7—C6	114.7 (2)	C27—C26—C36	118.9 (3)
C8—C7—C6	113.1 (2)	C27—C26—C25	122.9 (3)
C15—C7—H7	106.3	C36—C26—C25	118.1 (2)
C8—C7—H7	106.3	N4—C27—C26	128.3 (3)
C6—C7—H7	106.3	N4—C27—O3	110.0 (2)
C9—C8—C18	118.7 (3)	C26—C27—O3	121.7 (3)
C9—C8—C7	122.2 (2)	C33—C28—O3	122.5 (3)
C18—C8—C7	119.0 (2)	C33—C28—C29	126.2 (3)
N2—C9—C8	128.7 (3)	O3—C28—C29	111.3 (2)
N2—C9—O1	110.3 (2)	C28—C29—C30	113.0 (2)
C8—C9—O1	121.0 (2)	C28—C29—H29A	109.0
C15—C10—O1	122.5 (3)	C30—C29—H29A	109.0
C15—C10—C11	126.7 (3)	C28—C29—H29B	109.0
O1—C10—C11	110.8 (2)	C30—C29—H29B	109.0
C10—C11—C12	112.5 (3)	H29A—C29—H29B	107.8
C10—C11—H11A	109.1	C35—C30—C31	110.2 (3)
C12—C11—H11A	109.1	C35—C30—C34	108.6 (3)
C10—C11—H11B	109.1	C31—C30—C34	110.6 (3)
C12—C11—H11B	109.1	C35—C30—C29	109.8 (3)
H11A—C11—H11B	107.8	C31—C30—C29	107.9 (3)
C13—C12—C17	113.8 (3)	C34—C30—C29	109.7 (2)
C13—C12—C11	109.9 (3)	C32—C31—C30	112.9 (2)
C17—C12—C11	112.5 (3)	C32—C31—H31A	109.0
C13—C12—C16	108.5 (3)	C30—C31—H31A	109.0
C17—C12—C16	106.2 (3)	C32—C31—H31B	109.0
C11—C12—C16	105.5 (3)	C30—C31—H31B	109.0
C12—C13—C14	114.9 (3)	H31A—C31—H31B	107.8
C12—C13—H13A	108.5	O4—C32—C33	119.6 (3)
C14—C13—H13A	108.5	O4—C32—C31	121.8 (3)
C12—C13—H13B	108.5	C33—C32—C31	118.5 (3)
C14—C13—H13B	108.5	C28—C33—C32	117.5 (3)
H13A—C13—H13B	107.5	C28—C33—C25	123.3 (3)
O2—C14—C15	121.0 (3)	C32—C33—C25	119.1 (2)
O2—C14—C13	120.8 (3)	C30—C34—H34A	109.5
C15—C14—C13	118.0 (3)	C30—C34—H34B	109.5
C10—C15—C14	117.7 (3)	H34A—C34—H34B	109.5
C10—C15—C7	122.3 (3)	C30—C34—H34C	109.5
C14—C15—C7	119.9 (2)	H34A—C34—H34C	109.5
C12—C16—H16A	109.5	H34B—C34—H34C	109.5
C12—C16—H16B	109.5	C30—C35—H35A	109.5
H16A—C16—H16B	109.5	C30—C35—H35B	109.5
C12—C16—H16C	109.5	H35A—C35—H35B	109.5
H16A—C16—H16C	109.5	C30—C35—H35C	109.5
H16B—C16—H16C	109.5	H35A—C35—H35C	109.5
C12—C17—H17A	109.5	H35B—C35—H35C	109.5
C12—C17—H17B	109.5	N3—C36—C26	178.1 (3)
H17A—C17—H17B	109.5	C9—N2—H2A	120.0
C12—C17—H17C	109.5	C9—N2—H2B	120.0
H17A—C17—H17C	109.5	H2A—N2—H2B	120.0
H17B—C17—H17C	109.5	C27—N4—H4A	119.9
N1—C18—C8	178.4 (3)	C27—N4—H4B	120.1
C20—C19—C24	120.2 (4)	H4A—N4—H4B	120.0
C20—C19—Cl2	117.9 (3)	C9—O1—C10	119.6 (2)
C24—C19—Cl2	122.0 (2)	C28—O3—C27	118.7 (2)
C21—C20—C19	119.6 (4)	H1X—O1W—H1Y	109.5
C21—C20—H20	120.2		
			
C6—C1—C2—C3	−1.7 (5)	C21—C22—C23—C24	−0.7 (6)
Cl1—C1—C2—C3	179.2 (3)	C22—C23—C24—C19	0.1 (5)
C1—C2—C3—C4	1.9 (5)	C22—C23—C24—C25	−176.1 (3)
C2—C3—C4—C5	−1.4 (5)	C20—C19—C24—C23	0.6 (4)
C3—C4—C5—C6	0.7 (5)	Cl2—C19—C24—C23	179.4 (2)
C2—C1—C6—C5	1.0 (4)	C20—C19—C24—C25	176.8 (3)
Cl1—C1—C6—C5	−180.0 (2)	Cl2—C19—C24—C25	−4.4 (4)
C2—C1—C6—C7	−177.1 (3)	C23—C24—C25—C33	−54.9 (4)
Cl1—C1—C6—C7	1.9 (4)	C19—C24—C25—C33	129.0 (3)
C4—C5—C6—C1	−0.5 (4)	C23—C24—C25—C26	68.7 (4)
C4—C5—C6—C7	177.7 (3)	C19—C24—C25—C26	−107.4 (3)
C1—C6—C7—C15	−64.6 (4)	C33—C25—C26—C27	12.9 (4)
C5—C6—C7—C15	117.3 (3)	C24—C25—C26—C27	−113.5 (3)
C1—C6—C7—C8	61.8 (4)	C33—C25—C26—C36	−169.2 (3)
C5—C6—C7—C8	−116.3 (3)	C24—C25—C26—C36	64.3 (3)
C15—C7—C8—C9	18.8 (4)	C36—C26—C27—N4	−3.6 (5)
C6—C7—C8—C9	−110.4 (3)	C25—C26—C27—N4	174.2 (3)
C15—C7—C8—C18	−164.1 (3)	C36—C26—C27—O3	178.5 (3)
C6—C7—C8—C18	66.7 (3)	C25—C26—C27—O3	−3.7 (4)
C18—C8—C9—N2	−7.1 (5)	C33—C28—C29—C30	19.0 (4)
C7—C8—C9—N2	170.0 (3)	O3—C28—C29—C30	−161.9 (2)
C18—C8—C9—O1	173.3 (3)	C28—C29—C30—C35	−167.1 (3)
C7—C8—C9—O1	−9.6 (5)	C28—C29—C30—C31	−47.0 (3)
C15—C10—C11—C12	20.3 (5)	C28—C29—C30—C34	73.6 (3)
O1—C10—C11—C12	−161.7 (3)	C35—C30—C31—C32	175.0 (3)
C10—C11—C12—C13	−44.9 (4)	C34—C30—C31—C32	−64.9 (4)
C10—C11—C12—C17	−172.9 (3)	C29—C30—C31—C32	55.1 (4)
C10—C11—C12—C16	71.8 (4)	C30—C31—C32—O4	147.2 (3)
C17—C12—C13—C14	178.1 (3)	C30—C31—C32—C33	−35.1 (4)
C11—C12—C13—C14	50.9 (4)	O3—C28—C33—C32	−174.9 (3)
C16—C12—C13—C14	−63.9 (4)	C29—C28—C33—C32	4.1 (5)
C12—C13—C14—O2	154.8 (3)	O3—C28—C33—C25	1.2 (4)
C12—C13—C14—C15	−30.5 (5)	C29—C28—C33—C25	−179.8 (3)
O1—C10—C15—C14	−176.0 (3)	O4—C32—C33—C28	−178.1 (3)
C11—C10—C15—C14	1.8 (5)	C31—C32—C33—C28	4.1 (4)
O1—C10—C15—C7	1.4 (5)	O4—C32—C33—C25	5.6 (5)
C11—C10—C15—C7	179.2 (3)	C31—C32—C33—C25	−172.1 (3)
O2—C14—C15—C10	177.6 (3)	C26—C25—C33—C28	−11.7 (4)
C13—C14—C15—C10	2.9 (5)	C24—C25—C33—C28	113.1 (3)
O2—C14—C15—C7	0.2 (5)	C26—C25—C33—C32	164.3 (3)
C13—C14—C15—C7	−174.5 (3)	C24—C25—C33—C32	−70.9 (3)
C8—C7—C15—C10	−14.7 (4)	C27—C26—C36—N3	−117 (10)
C6—C7—C15—C10	113.6 (3)	C25—C26—C36—N3	65 (10)
C8—C7—C15—C14	162.6 (3)	N2—C9—O1—C10	174.3 (3)
C6—C7—C15—C14	−69.1 (4)	C8—C9—O1—C10	−6.0 (5)
C9—C8—C18—N1	−92 (12)	C15—C10—O1—C9	10.3 (5)
C7—C8—C18—N1	91 (12)	C11—C10—O1—C9	−167.8 (3)
C24—C19—C20—C21	−0.8 (5)	C33—C28—O3—C27	9.9 (4)
Cl2—C19—C20—C21	−179.6 (3)	C29—C28—O3—C27	−169.2 (2)
C19—C20—C21—C22	0.2 (6)	N4—C27—O3—C28	173.2 (3)
C20—C21—C22—C23	0.5 (6)	C26—C27—O3—C28	−8.6 (4)

### Hydrogen-bond geometry (Å, º)

**Table:**

*D*—H···*A*	*D*—H	H···*A*	*D*···*A*	*D*—H···*A*
N2—H2*A*···N3^i^	0.86	2.11	2.957 (4)	167
N2—H2*B*···O1*W*^ii^	0.86	2.01	2.793 (4)	152
N4—H4*A*···N1^iii^	0.86	2.20	3.048 (4)	169
N4—H4*B*···O2^iv^	0.86	2.13	2.957 (3)	161
O1*W*—H1*X*···O4	0.96	1.95	2.754 (4)	139
O1*W*—H1*Y*···Cl2	0.96	2.29	3.168 (4)	151

Symmetry codes: (i) *x*, −*y*, *z*+1/2; (ii) −*x*+2, *y*, −*z*+3/2; (iii) *x*, −*y*, *z*−1/2; (iv) −*x*+3/2, −*y*+1/2, −*z*+1.
